# Empagliflozin in kidney transplant recipients with chronic kidney disease G3a-4 and metabolic syndrome: Five Japanese cases

**DOI:** 10.1186/s12882-022-02793-9

**Published:** 2022-05-02

**Authors:** Ryoichi Miyazaki, Kyoko Miyagi

**Affiliations:** Department of Internal Medicine, Fujita Memorial Hospital, 4-15-7, Fukui, Fukui 910-00004 Japan

**Keywords:** Renal transplant recipient, Empagliflozin, Stage G3b chronic kidney disease

## Abstract

**Background:**

Sodium-glucose cotransporter 2 (SGLT2) inhibitors have been shown to exert cardiorenal protective effects in diabetic patients and are widely used clinically. In addition, an increasing number of reports now suggest these drugs may even be beneficial in non-diabetic patients. However, SGLT2 inhibitors are rarely prescribed for kidney transplant recipients due to the risk of renal graft damage and urogenital infections.

**Case presentation:**

We report the cases of 5 renal transplant recipients with chronic kidney disease G3a-4 and metabolic syndrome who were administered the SGLT2 inhibitor empagliflozin, which yielded beneficial results in 4 cases. With the exception of one patient with an initial estimated glomerular filtration rate (eGFR) of less than 30 ml/min/1.73 m2, administration of empagliflozin elicited beneficial metabolic effects. There were no significant reductions in eGFR before or after empagliflozin administration, and no dehydration or urogenital infections were observed during the treatment course.

**Conclusion:**

Empagliflozin showed some positive effects in 4 cases with better renal function than CKD stage 4. Further studies will be required to clarify the efficacy and safety of SGLT2 inhibitors in a larger group of patients with similar medical conditions.

## Background

Post-transplantation diabetes mellitus (PTDM) refers to diabetes present after transplantation, irrespective of the timing of the diagnosis or whether it was present but undetected before transplantation [1]. According to a large-scale survey from the United States, PTDM occurs in about 9%, 16%, and 24% of patients, respectively, 3 months, 1 year, and 3 years after transplantation [2]. Among kidney transplant patients, the incidence of PTDM is reportedly 5.9% 6 months after transplantation, 7.1% after 1 year, 10.4% after 3 years, 13.2% after 5 years and 20.5% after 10 years [3]. This is noteworthy, as mortality during an 8-year follow-up period after kidney transplantation was reportedly 16% among non-diabetic patients but 22% among diabetic patients [4]. PTDM is also reported to be an independent risk factor for decreased renal transplant function [5]. Insulin is often required as treatment for PTDM. Metformin and sulfonylureas are the most frequently used medical treatments for PTDM. Dipeptidyl peptidase-4 (DPP-4) inhibitors, which can be used relatively safely in patients with chronic kidney disease (CKD), are also increasingly used. [6]. Sodium-glucose transport protein (SGLT2) inhibitors have been shown to exert renal and cardioprotective effects in large-scale clinical trials, and their usefulness has been well established [7–11]. However, Sodium-glucose cotransporter 2 (SGLT2) inhibitors are rarely prescribed for PTDM. The reason is that there is a risk of renal dysfunction and urinary tract infection due to dehydration caused by these drugs [12]. For this reason, there is little literature on the use of SGLT2 inhibitors for PTDM, and they are rarely used [6].

## Case presentation

**Fig. 1:**
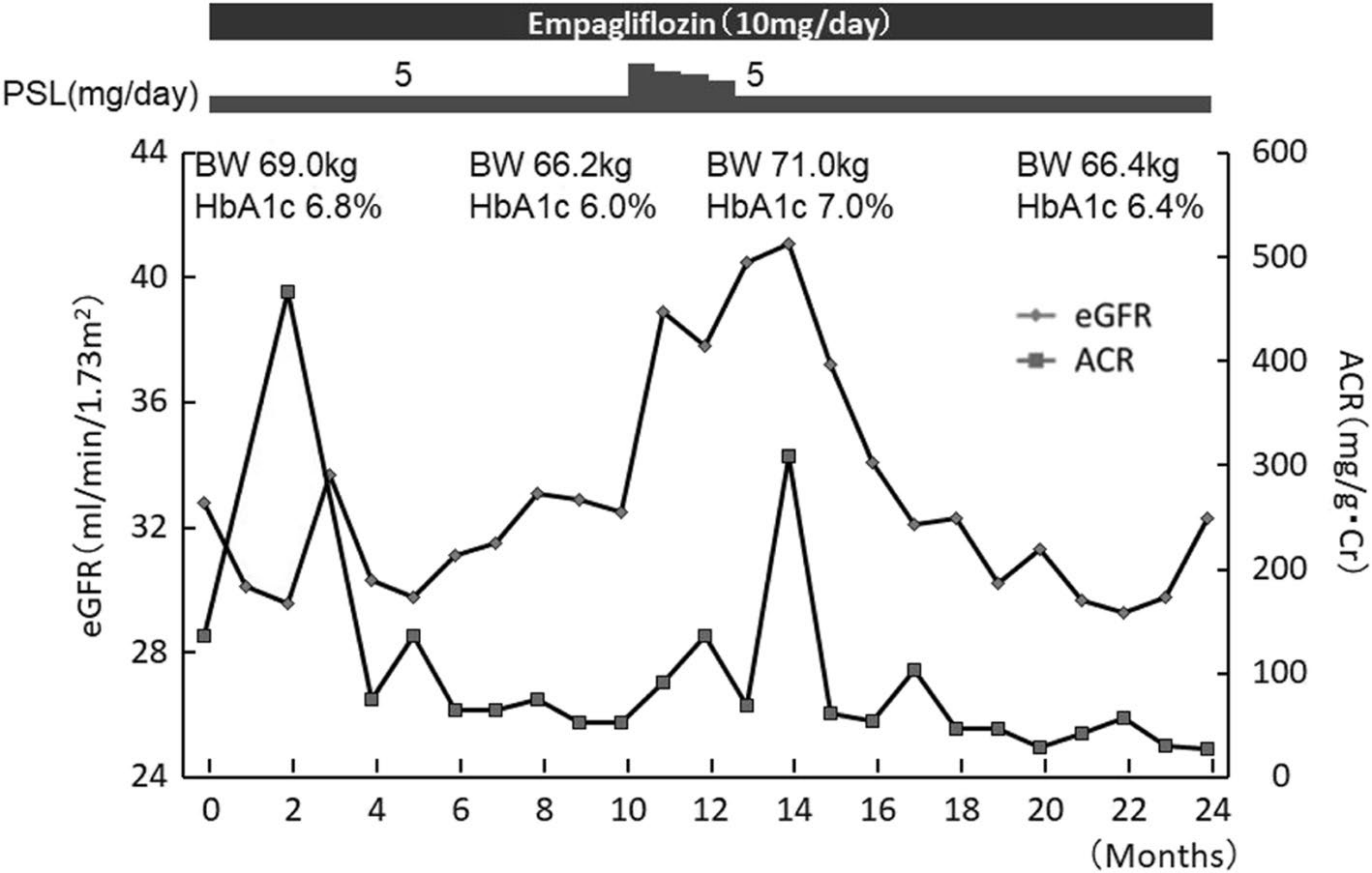
Clinical course of Case1. In case 1, HbA1c and UACR increased and decreased with changes in body weight. There was no change in renal function during the course.

Here we present the results from 5 patients treated with empagliflozin for metabolic syndrome following living-donor kidney transplantation (Table1). The age range was 51-68 years, and the time after transplantation was 4.3-24 years. Immunosuppressive drugs, other medications, and complications during administration are shown in Table 1. Steroids and calcineurin inhibitors were given to all patients. The calcineurin inhibitor was cyclosporine A in 3 cases and tacrolimus in 2 cases. All 5 patients were male and met the Japanese diagnostic criteria of an abdominal circumference of 85 cm or more for men, 90cm or more for women plus 2 or more of the following elevated TG and/or reduced HDL, elevated BP, elevated FPG. [13] with two or more other risk factors (Table 2). All 5 patients were administered empagliflozin 10 mg/day in addition to the other prescriptions. The subsequent observation period ranged from 8 to 24 months. Before and after empagliflozin administration, body weight decreased in 2 cases, increased in 1 case, and remained unchanged in the other 2 cases. Waist circumference changed in parallel with body weight. Systolic blood pressure decreased in 2 cases, increased slightly in 1 case, and remained unchanged in the other 2 cases. The changes in diastolic blood pressure were similar to those in systolic blood pressure. Serum Cr increased slightly in 3 cases and decreased slightly in 2 cases. Changes in eGFR corresponded to the changes in serum Cr. In case 2, alanine aminotransferase (ALT) was high before empagliflozin administration, but decreased slightly after administration. The other 4 cases showed normal values, which were ​​unchanged by empagliflozin administration. Changes in gamma-glutamyl transpeptidase (γ-GTP) were similar to the changes in ALT. Triglyceride (TG) decreased in 3 cases and was unchanged in the other 2 cases. Hemoglobin A1c (HbA1c) levels decreased in 3 cases and remained unchanged in 2 cases. The urine albumin creatinine ratio (UACR) decreased in 3 cases and remained unchanged in the other 2 cases. Figure 1 shows the clinical course of case 1 during empagliflozin administration. Following a transient increase, UACR decreased to near normal. However, after 10 months of empagliflozin administration, polymyalgia rheumatica-like symptoms appeared. When the prednisolone (PSL) dose was then increased from 5 mg/day to 15 mg/day, body weight and HbA1c increased with the increase in appetite, and UACR was also elevated. Polymyalgia rheumatica was subsequently ruled out, and PSL was reduced to the original 5 mg/day. With the subsequent reduction in body weight, HbA1c declined and eventually UACR also decreased to a level that was lower than the level prior to empagliflozin administration. Table 2 shows the clinical course of the five patients before and after SGLT2 inhibitor administration. To summarize, there was no significant decrease in renal function with administration of empagliflozin. With the exception of case 3, each case exhibited beneficial changes with empagliflozin administration. In case 1, body weight, blood pressure, and HbA1c all decreased, and the elevated UACR decreased to near normal. In case 2, blood pressure, ALT, γ-GTP, and TG all decreased, and UACR normalized. In case 4, body weight, blood pressure, and HbA1c decreased. In case 5, body weight, uric acid, TG, and HbA1c all decreased, and UACR was also slightly reduced. In cases 1 and 2, however, weight rebound was observed. In the future, such cases will require nutrition and exercise guidance. During the treatment course, no patient developed acute kidney injury or a urogenital infection.

**Table 1:** Clinical profiles of the patients before SGLT-2 inhibitor administration

	Case 1	Case 2	Case 3	Case 4	Case 5
Gender	Male	Male	Male	Male	Male
Age(years)	68	57	60	56	51
Primary renal disease	IgAN	IgAN	IgAN	DM	CGN
Period after transplantation (years)	23.7	20.5	24.0	4.9	4.3
Waist circumference (cm)	92	104	91	94	112
Immunosuppressant	PSL, HCZ, CyA MZB	PSL, CyA MZB, EVR	PSL, CyA MMF, EVR	PSL, TAC MMF, EVR	PSL, TAC MZB, EVR
Concomitant drugs	Febuxostat Levothyroxine	Febuxostat Fluvastatin Cilnidipine Valsartan	Febuxostat Fluvastatin Cilnidipine Valsartan	Nifedipine Valsartan Dulaglutide Insulin degludec	Febuxostat Fluvastatin Ezetimibe
Complications	MetS, PTDM, HT HU, PsV, SAI Hypothyroidism	MetS, HT,HU Fatty liver Dyslipidemia	MetS, HT, HU Dyslipidemia	MetS, DM, HT	MetS, PTDM, HT Dyslipidemia C9 deficiency
Family history	DM(-) Renal disease(-)	DM(-) Renal disease(-)	DM(-) Renal disease(-)	DM(-) Renal disease(-)	DM(-) Renal disease(-)
Past history	Pneumonia 10 times	Hopes zoster	Hypouricemia	None	Gastroduodenal ulcer

Abbreviations: *BP* blood pressure, *CGN* chronic glomerulonephritis, *CyA* cyclosporin A, *DM* diabetes mellitus, *EVR* everolimus, *HCZ* hydrocortisone, *HT* hypertension, *HU* Hyperuricemia *IgAN* IgA nephropathy, *IGT* impaired glucose tolerance, *MetS* metabolic syndrome, *MMF* mycophenolate mofetil, *MZB* mizoribine, *PTDM* post-transplant diabetes mellitus *PsV* psoriasis vulgaris, *SAI* secondary adrenal insufficiency, *TAC* tacrolimus

**Table 2:** Clinical course before and after SGLT-2 inhibitor administration

	Case 1	Case 2	Case 3	Case 4	Case 5
Body weight (Kg)	69.0/67.8	73.4/83.3	73.4/73.3	89.1/86.2	99.9/98.2
Waist circumference (cm)	92/90	104/106	91/89	94/91	112/111
SBP (mmHg)	159/103	140/124	132/131	135/125	126/139
SBP (mmHg)	79/60	72/66	76/78	79/77	85/87
Cr (mg/dL)	1.69/1.71	1.40/1.70	2.16/2.37	1.33/1.25	1.89/1.67
eGFR (ml/min/1.73^2^)	32.8/32.1	39.5/33.9	25.9/23.4	45.0/47.9	32.4/36.0
UA(mg/dL)	5.1/5.4	4.2/4.0	4.4/3.9	5.5/5.8	4.1/5.5
ALT (IU/L)	9/8	73/51	16/9	26/21	39/36
γ-GTP (IU/L)	11/9	89/58	57/40	30/24	40/37
TG (mg/dL)	121/116	188/133	248/248	112/76	173/99
HbA1c (%)	6.8/6.4	6.0/5.9	5.5/5.6	7.6/7.1	6.0/5.8
UACR (mg/gCr)	135.7/27.7	35.8/10.8	790.0/650.0	24.0/19.1	46.9/23.6
Observation period (months)	24	18	18	9	8

Values show data before/after empagliflozin administration.

Abbreviations: *ALT* alanine aminotransferase, Cr creatinine, *γ-GTP* γ-glutamyl transpeptidase, *DBP* diastolic blood pressure, *eGFR* estimated glomerular filtration rate, *HbA1c* hemoglobin A1c, *SBP* systolic blood pressure, *TG* triglyceride, *UA* uric acid, *UACR* urinary albumin-to-creatinine ratio

This case report has several limitations. First, this report is a single-center study with a small number of cases. The fact that all patients were male was considered a factor in the absence of urinary tract infections. The observation period was short, ranging from 8 to 24 months.

## Discussion

PTDM is defined as a post-transplant diabetic condition, regardless of the timing of diabetes onset [1]. Long-term glucose management of PTDM frequently requires insulin over time, particularly in those with the greatest obesity. Metformin is often the first-line drug for treatment of PTDM. However, metformin is contraindicated in patients with eGFR less than 30 mL/min/1.73 m3 because of the risk of lactic acidosis. Sulfonylureas are commonly utilized for PTDM, with little available safety or efficacy data. Dipeptidyl-peptidase-4(DPP-4) inhibitors have a relatively low risk of hypoglycemia, are weight neutral, and can be used safely in patients who have only mild reductions in kidney

function or if the dose is adjusted appropriately with more significant chronic kidney disease. Because of these factors, as well as evidence that they do not affect immunosuppressant levels, DPP-4 inhibitors are increasingly used for treatment of PTDM without significant safety concerns identified. [6]. SGLT2 inhibitors not only lower blood sugar, in recent large-scale studies they were shown to protect both the heart and kidneys. For example, empagliflozin was first shown to protect the heart and kidneys of non-transplant patients (EMPA-REG Outcome study [7]), and similar benefits were observed in the CANVAS [8] and DECLARE-TIMI58 trials [9]. The CREDENCE trial [10], which had renal protection as an outcome, also demonstrated the efficacy of empagliflozin. In the DAPA-HF study [5], the efficacy of another SGLT2 inhibitor, dapagliflozin, was observed in patients with heart failure with reduced left ventricular ejection fraction, with and without diabetes. The most important mechanism by which SGLT2 inhibitors protect the kidneys is restoration of tubuloglomerular feedback (TGF), which causes pre-glomerular vasoconstriction. In diabetic patients, elevated blood glucose and glomerular hyperfiltration increase urinary glucose excretion, resulting in enhanced SGLT2-mediated glucose reabsorption in the renal tubules. Along with glucose, the reabsorption of Na and Cl also increases. As a result, the amount of Na and Cl reaching the distal convoluted tubules is reduced, and that information is transmitted to the glomerulus via the macula densa (i.e., TGF), resulting in pre-glomerular vasodilatation and elevated intraglomerular pressure. Administration of an SGLT2 inhibitor suppresses the reabsorption of Na and Cl as well as glucose in the renal tubules, resulting in restored TGF. It is said that this restoration in TGF enhances pre-glomerular vasoconstriction and reduces the intraglomerular pressure, which helps to protect the kidneys [14]. Other mechanisms contributing to the renal protective action of SGLT2 inhibitors are thought involve elevation of ketone bodies [15] and reduction of oxidative stress [16].

Despite the efficacy of SGLT2 inhibitors in non-transplanted patients, administration of SGLT2 inhibitors to renal transplant patients remains controversial [6, 17, 18]. Shivaswamy et al. [6] have expressed concerns about potential genitourinary infections and decreased renal function related to dehydration. They suggested that SGLT2 inhibitors should be avoided in renal transplant recipients until their safety is better established. The main findings of the SGLT2 inhibitor trials in KTR we searched are summarized in Table 3 [19–23]. These 5 studies explored the effects of SGLT2 inhibitors in renal transplant recipients with CKD stage 1-3b, where renal function was relatively preserved. However, results from several other reports suggest SGLT2 inhibitors may also be useful in patients with more severe renal damage [24–29]. Although SGLT inhibitors were previously thought to be ineffective in advanced CKD patients [26], there is increasing evidence showing their efficacy. Yamout et al. [24] compared 338 patients receiving canagliflozin at 100 mg/day and 365 patients receiving 300 mg/day with 382 patients receiving placebo. In each group the eGFR averaged about 40 mL/min/1.73 m^2^, and both doses significantly decreased HbA1c, body weight and systolic blood pressure as compared to placebo. In 5 other studies [25–29], SGLT2 inhibitors were reported to have similar effects in patients with stage 3-4 CKD.

**Table 3:** Summary of reports on SGLT2 inhibitor use in diabetic renal transplant patients

Number of cases	Age (years) mean±SD or (range)	Sex male/female	eGFR (ml/min/1.73 m^2^) Baseline Changes after SGLT2 inhibitor	HbA1c (%) Baseline Changes after SGLT2 inhibitor	Systolic blood pressure (mmHg) Baseline Changes after SGLT2 inhibitor	Diastolic blood pressure (mmHg) Baseline Changes after SGLT2 inhibitor	Body weight (kg) Baseline Changes after SGLT2 inhibitor	References
14	56.5±7.9	7/7	54.0±23.8 53.5±13.3	6.7±0.7 7.1±0.8	150±26 145±20	86±14 76±11	83.7±7.6 78.7±7.7	[19]
22	63 (31-72)	17/5	66(57-68) 59(52-67)	6.9(6.5-8.2) 6.9(6.4-7.4)	143(111-176) 140(100-163)	76(71-82) 80(74-86)	92.0(81.8-104.5) 85.0(79.5-97.5)	[20]
4 (SPKTR)	49.4±8.9	2/2	60±14 -4.3±12.2^a^	7.4±1.1 *-0.84±1.2	not provided -6.5±10.8^a^	not provided -4.8±12^a^	not provided -2.14±2.8^a^	[21]
6 (KTR)	61.±12.6	5/1	78±18.2 -4.3±12.2^a^	8.6±1.4 -0.84^a^	not provided -6.5±10.8^a^	not provided -4.8±12^a^	not provided -2.14±2.8^a^	
24	53.8±7.1	23/1	Ccr 86±20 83±18	8.5±1.5 7.6±1.0	142±21 134±17	81±9 79±8	78.6±12.1 76.2±10.9	[22]
8	56.8±13.7	6/2	75.75±13.38 69.88±14.70	9.34±1.36 7.41±1.44	135±9.59 126.43±11.46	80.63±10.13 74.75±7.25	BMI 32.74±7.2 27.4±4.2	[23]

Abbreviations: SPKTR, simultaneous pancreas-kidney transplant recipients; KTR, kidney transplant recipients

^*^amount of change in both the SPKTR and KTR group; mean±SD

In case 3 in the present study, the patient had an eGFR of 25.9 ml/min/1.73m^2^ (CKD stage 4) before empagliflozin administration, and no useful effect of this drug was observed. In this case, the excretion of urinary sugar induced by empagliflozin was considered to be insufficient due to advanced renal dysfunction, and no good effect was obtained. In the other 4 cases, however, empagliflozin administration led to weight loss, decreased blood pressure, decreased ALT or γ-GTP, decreased HbA1c, and/or decreased UACR. In case 2, the patient exhibited decreases in both ALT and γ-GTP after empagliflozin administration. We suggest that these decreases in ALT and γ-GTP were due to improvement in this patient’s fatty liver disease [30].

The only randomized clinical trial (RCT) of SGTL2 inhibitor treatment in patients with PTDM is Halden et al [20]. They compared 22 empagliflozin-treated patients with 22 placebo-treated patients. The results showed that empagliflozin-treated patients had significantly HbA1c, uric acid and body weight reduction, and than placebo-treated patients. There was no change in eGFR before or after SGLT2 inhibitor treatment in the empagliflozin group. Three cases of urinary tract infection were seen in each group, and one case of urosepsis in the empagliflozin group was discontinued. Other adverse events were minor and did not differ between the two groups. In our case, diabetes mellitus was present in three of the five cases, and the other two cases had metabolic syndrome but not diabetes mellitus. Therefore, in the three diabetic patients, HbA1c decreased. But in the two patients with metabolic syndrome, HbA1c was relatively low before empagliflozin administration and did not change after the drug was administered. With regard to uric acid, febuxostat was administered in 4 of the 5 patients, and serum uric acid levels were low at the time of drug initiation, and no pre- or post-dose changes were observed overall. In the present case, weight decreased in three of the five diabetic patients and increased or remained unchanged in the other two patients with metabolic syndrome.

Recently, Jenssen reviewed the efficacy and safety of SGLT2 inhibitors in patients with PTDM [18]. Jenssen noted that precautions for SGLT2 inhibitors in patients with PTDM include genitourinary tract infections, ketoacidosis and decreased renal function, including acute kidney injury (AKI) due to dehydration associated with increased urinary glucose. He summarized that urinary tract infections were not more frequent in KTR than in other patients who received SGLT2 inhibitors, and that bacterial genital infections were in the range expected in KTR in all studies and also seen in patients with type 2 diabetes.

## Conclusion

In this report, we describe the efficacy and adverse effects of empagliflozin administered to 5 Japanese patients with CKD G3a-4 and metabolic syndrome after renal transplantation. Empagliflozin showed beneficial effects in 4 patients with better renal function than CKD 4. No adverse events, including deterioration of renal function, were observed in any patient. The number of cases in the literature so far is small, ranging from 8 to 22, and we believe that RCTs with a larger number of patients are needed to determine the efficacy and safety of SGLT2 inhibitors in KTR. Our case series is also small, only five cases, but there are still very few reports worldwide, and we hope that this presentation will trigger further research.

## Data Availability

We cannot share the data, because we have no data beyond what is presented in Tables and Figure.

## Abbreviations

AKI: Acute Kidney Injury
ALT: Alanine Aminotransferase
BP: Blood Pressure
CGN: Chronic Glomerulonephritis
CKD: Chronic Kidney Disease
Cr: Creatinine
CyA: Cyclosporin A
DBP: Diastolic Blood Pressure
DM: Diabetes Mellitus
DPP4: Dipeptidyl-peptidase-4
eGFR: estimated Glomerular Filtration Rate
EVR: Everolimus
γ-GTP: Gamma-glutamyl Transpeptidase
GLP-1: Glucagon-like Peptide-1
HbA1c: Hemoglobin A1c
HCZ: Hydrocortisone
HT: Hypertension
HU: Hyperuricemia
IgAN: IgA Nephropathy
IGT: Impaired Glucose Tolerance
KTR: Kidney Transplant Recipients
MetS: Metabolic Syndrome
MMF: Mycophenolate Mofetil MZB, Mizoribine
PSL: Prednisolone
PsV: Psoriasis Vulgaris
PTDM: Post-transplant Diabetes Mellitus
RCT: Randomized Clinical Trial
SAI: Secondary Adrenal Insufficiency
SBP: Systolic Blood Pressure
SGLT2: Sodium-glucose Transporter 2
SPKTR: Simultaneous Pancreas-kidney Transplant Recipients
TAC: Tacrolimus
TG: Triglyceride
UACR: Urine Albumin Creatinine Ratio
